# Supplementary material for: Smoking in social housing among adults in England, 2015–2020: a nationally representative survey
Source: BMJ Open. 2022 Jul 26;12(7):e061013. doi: 10.1136/bmjopen-2022-061013 (PMC9330323; doi:10.1136/bmjopen-2022-061013)
Supplement: Supplementary data [file bmjopen-2022-061013supp001.pdf]

Smoking in social housing among adults in England, 2015-2020: a nationally representative survey

Sarah E. Jackson[1,2], Hazel Cheeseman[3], Deborah Arnott[3], Robbie Titmarsh[3], Jamie Brown[1,2]

[1] Department of Behavioural Science and Health, University College London, London, UK

[2] SPECTRUM Consortium, UK

[3] Action on Smoking and Health, London, UK

Supplementary material

Supplementary Table 1. Interactions between housing tenure and age group

Supplementary Table 2. Interactions between housing tenure and sex

Supplementary Table 3. Association between use of evidence-based support and cessation among past-year smokers who made a quit attempt: interaction with housing tenure

Supplementary Table 4. Sensitivity analysis using log-binomial regression to test associations between housing tenure and smoking and cessation behaviour

Supplementary Table 1. Interactions between housing tenure and age group

	35-64 (vs. 16-34)			≥65 (vs. 16-34)		
	OR/B*	95% CI	*p*	OR/B	95% CI	*p*
All adults						
% Cigarette smokers	1.42	1.30 to 1.55	<0.001	1.37	1.20 to 1.56	<0.001
Current cigarette smokers						
Mean cigarettes per day	-0.97	-1.53 to -0.41	0.001	-1.86	-2.75 to -0.96	<0.001
% First smoke within 30 min of waking	0.86	0.74 to 1.00	0.043	0.59	0.47 to 0.74	<0.001
% High motivation to stop	1.00	0.82 to 1.22	0.994	0.90	0.61 to 1.34	0.611
% Regular exposure to smoking by others	1.22	1.04 to 1.43	0.015	1.48	1.17 to 1.88	0.001
Past-year smokers						
% Past year quit attempt	1.15	0.99 to 1.33	0.069	1.02	0.78 to 1.32	0.912
Past year quit attempt						
% Not currently smoking	0.92	0.64 to 1.32	0.645	1.14	0.60 to 2.17	0.682
% Used any cessation support**	0.77	0.60 to 0.99	0.037	0.79	0.50 to 1.24	0.299
% Used behavioural support	0.62	0.26 to 1.49	0.284	0.37	0.11 to 1.24	0.107
% Used NRT OTC	0.74	0.51 to 1.07	0.112	1.17	0.64 to 2.12	0.616
% Used e-cigarettes	0.84	0.65 to 1.08	0.177	0.73	0.42 to 1.26	0.261
% Used prescription medication	0.98	0.60 to 1.60	0.930	0.83	0.39 to 1.79	0.637

* OR/B adjusted for sex, age, social grade, government office region, and survey year. **Any cessation support includes behavioural support, nicotine replacement therapy (NRT) bought over-the-counter (OTC), e-cigarettes, and prescription medication.

Supplementary Table 2. Interactions between housing tenure and sex

	Female (vs. male)		
	OR/B*	95% CI	*p*
All adults			
% Cigarette smokers	1.11	1.02 to 1.20	0.014
Current cigarette smokers			
Mean cigarettes per day	-0.11	-0.64 to 0.42	0.678
% First smoke within 30 min of waking	1.02	0.89 to 1.17	0.805
% High motivation to stop	0.90	0.74 to 1.09	0.283
% Regular exposure to smoking by others	0.93	0.80 to 1.07	0.302
Past-year smokers			
% Past year quit attempt	0.97	0.84 to 1.11	0.640
Past year quit attempt			
% Not currently smoking	1.02	0.72 to 1.45	0.901
% Used any cessation support**	1.23	0.97 to 1.56	0.093
% Used behavioural support	0.82	0.39 to 1.74	0.605
% Used NRT OTC	1.13	0.80 to 1.61	0.486
% Used e-cigarettes	1.14	0.89 to 1.47	0.308
% Used prescription medication	1.13	0.74 to 1.74	0.571

* OR/B adjusted for sex, age, social grade, government office region, and survey year. **Any cessation support includes behavioural support, nicotine replacement therapy (NRT) bought over-the-counter (OTC), e-cigarettes, and prescription medication.

Supplementary Table 3. Association between use of evidence-based support and cessation among past-year smokers who made a quit attempt: interaction with housing tenure

	Not currently smoking		
	OR*	95% CI	*p*
Used any cessation support**	1.11	0.94-1.30	0.218
Housing tenure	0.62	0.46-0.84	0.002
Used any cessation support x housing tenure (interaction)	0.93	0.64-1.34	0.684

*OR adjusted for sex, age, social grade, government office region, survey year, mean cigarettes per day, and smoking first cigarette within 30 minutes of waking.

**Any cessation support includes behavioural support, nicotine replacement therapy (NRT) bought over-the-counter (OTC), e-cigarettes, and prescription medication.

Supplementary Table 4. Sensitivity analysis using log-binomial regression to test associations between housing tenure and smoking and cessation behaviour

	Social housing	Other housing	Unadjusted			Adjusted*		
			RR	95% CI	*p*	RR	95% CI	*p*
**All adults**								
% Cigarette smokers	33.5	14.8	2.29	2.21 to 2.37	<0.001	1.73	1.66 to 1.80	<0.001
**Current cigarette smokers**								
% First smoke within 30 min of waking	57.4	42.6	1.34	1.27 to 1.42	<0.001	1.20	1.13 to 1.28	<0.001
% High motivation to stop	14.7	15.0	0.97	0.89 to 1.07	0.555	1.02	0.93 to 1.13	0.689
% Regular exposure to smoking by others	68.4	68.6	1.01	0.96 to 1.06	0.854	1.01	0.96 to 1.07	0.687
**Past-year smokers**								
% Past-year quit attempt	32.4	30.9	1.06	0.99 to 1.13	0.088	1.11	1.03 to 1.19	0.005
**Past-year quit attempt**								
% Not currently smoking	11.6	18.9	0.62	0.52 to 0.73	<0.001	0.69	0.58 to 0.82	<0.001
% Used any cessation support**	59.0	54.4	1.10	1.00 to 1.20	0.051	1.09	0.99 to 1.21	0.086
% Used behavioural support	2.8	2.2	1.27	0.90 to 1.79	0.180	1.27	0.86 to 1.87	0.237
% Used NRT OTC	13.4	13.0	1.06	0.90 to 1.24	0.507	0.91	0.76 to 1.09	0.305
% Used e-cigarettes	33.9	32.1	1.06	0.95 to 1.18	0.319	1.11	0.98 to 1.25	0.103
% Used prescription medication	9.0	7.1	1.28	1.05 to 1.56	0.015	1.33	1.06 to 1.66	0.014

*RR adjusted for sex, age, social grade, government office region, and survey year.

**Any cessation support includes behavioural support, nicotine replacement therapy (NRT) bought over-the-counter (OTC), e-cigarettes, and prescription medication.
