# Supplementary material for: Smoking in social housing among adults in England, 2015–2020: a nationally representative survey
Source: BMJ Open. 2022 Jul 26;12(7):e061013. doi: 10.1136/bmjopen-2022-061013 (PMC9330323; doi:10.1136/bmjopen-2022-061013)
Supplement: Author's manuscript [file bmjopen-2022-061013.draft_revisions.pdf]

BMJ Open is committed to open peer review. As part of this commitment we make the peer review history of every article we publish publicly available.

When an article is published we post the peer reviewers' comments and the authors' responses online. We also post the versions of the paper that were used during peer review. These are the versions that the peer review comments apply to.

The versions of the paper that follow are the versions that were submitted during the peer review process. They are not the versions of record or the final published versions. They should not be cited or distributed as the published version of this manuscript.

BMJ Open is an open access journal and the full, final, typeset and author-corrected version of record of the manuscript is available on our site with no access controls, subscription charges or pay-per-view fees (http://bmjopen.bmj.com).

If you have any questions on BMJ Open's open peer review process please email info.bmjopen@bmj.com

BMJ Open

Smoking in social housing among adults in England, 2015-2020: a nationally representative survey

Journal:	*BMJ Open*
Manuscript ID	bmjopen-2022-061013
Article Type:	Original research
Date Submitted by the Author:	09-Feb-2022

Complete List of Authors:	Jackson, Sarah ; UCL, Behavioural Science and Health<br>Cheeseman, Hazel; Action on Smoking and Health<br>Arnott, Deborah; Action on Smoking and Health<br>Titmarsh, Robbie; Action on Smoking and Health<br>Brown, Jamie; UCL, Behavioural Science and Health

Keywords:	EPIDEMIOLOGY, PUBLIC HEALTH, GENERAL MEDICINE (see Internal Medicine)

SCHOLARONE™
Manuscripts

I, the Submitting Author has the right to grant and does grant on behalf of all authors of the Work (as defined in the below author licence), an exclusive licence and/or a non-exclusive licence for contributions from authors who are: i) UK Crown employees; ii) where BMJ has agreed a CC-BY licence shall apply, and/or iii) in accordance with the terms applicable for US Federal Government officers or employees acting as part of their official duties; on a worldwide, perpetual, irrevocable, royalty-free basis to BMJ Publishing Group Ltd ("BMJ") its licensees and where the relevant Journal is co-owned by BMJ to the co-owners of the Journal, to publish the Work in this journal and any other BMJ products and to exploit all rights, as set out in our licence.

The Submitting Author accepts and understands that any supply made under these terms is made by BMJ to the Submitting Author unless you are acting as an employee on behalf of your employer or a postgraduate student of an affiliated institution which is paying any applicable article publishing charge ("APC") for Open Access articles. Where the Submitting Author wishes to make the Work available on an Open Access basis (and intends to pay the relevant APC), the terms of reuse of such Open Access shall be governed by a Creative Commons licence – details of these licences and which Creative Commons licence will apply to this Work are set out in our licence referred to above.

Other than as permitted in any relevant BMJ Author's Self Archiving Policies, I confirm this Work has not been accepted for publication elsewhere, is not being considered for publication elsewhere and does not duplicate material already published. I confirm all authors consent to publication of this Work and authorise the granting of this licence.

For peer review only

Smoking in social housing among adults in England, 2015-2020: a nationally representative survey

Sarah E. Jackson[1,2], Hazel Cheeseman[3], Deborah Arnott[3], Robbie Titmarsh[3], Jamie Brown[1,2]

[1] Department of Behavioural Science and Health, University College London, London, UK

[2] SPECTRUM Consortium, UK

[3] Action on Smoking and Health, London, UK

Corresponding author: Dr Sarah Jackson, Department of Behavioural Science and Health, University College London, 1-19 Torrington Place, London WC1E 7HB, UK. s.e.jackson@ucl.ac.uk +44 (0)207 679 8312

Word count: 1909

Number of tables: 2

Number of figures: 1

For peer review only - http://bmjopen.bmj.com/site/about/guidelines.xhtml

Abstract

Objectives: To analyse associations between living in social housing and smoking in England and evaluate progress toward reducing disparities in smoking prevalence among residents of social housing compared with other housing types.

Design: Nationally-representative, cross-sectional survey between January 2015 and February 2020.

Setting: England.

Participants: 105,562 adults (≥16y).

Primary and secondary outcome measures: Linear and logistic regression were used to analyse associations between living in social housing (vs. other housing types) and smoking status, cigarettes per day, time to first cigarette, exposure to smoking by others, motivation to stop smoking, quit attempts, and use of cessation support. Analyses adjusted for sex, age, social grade, region, and survey year.

Results: Adults living in social housing had twice the odds of being a smoker (OR_{adj}=2.17, 95%CI 2.08-2.27), and the decline in smoking prevalence between 2015 and 2020 was less pronounced in this high-risk group (-7%; OR_{adj}=0.98, 95%CI 0.96-1.01) than among adults living in other housing types (-24%; OR_{adj}=0.95, 95%CI 0.94-0.96; housing tenure*survey year interaction *p*=0.020). Smokers living in social housing were more addicted than those in other housing (smoking within 30 minutes of waking: OR_{adj}=1.50, 95%CI 1.39-1.61), but were no less motivated to stop smoking (OR_{adj}=1.06, 95%CI 0.96-1.17) and had higher odds of having made a serious attempt to quit in the past year (OR_{adj}=1.16, 95%CI 1.07-1.25). Among smokers who had tried to quit, those living in social housing had higher odds of using evidence-based cessation support (OR_{adj}=1.22, 95%CI 1.07-1.39) but lower odds of remaining abstinent (OR_{adj}=0.63, 95%CI 0.52-0.76).

Conclusions: There remain stark inequalities in smoking and quitting behaviour by housing tenure in England, with declines in prevalence stalling between 2015 and 2020 despite progress in the rest of the population. In the absence of targeted interventions to boost quitting among social housing residents, inequalities in health are likely to worsen.

Key words: smoking; social housing; housing tenure; inequalities

For peer review only - http://bmjopen.bmj.com/site/about/guidelines.xhtml

Strengths and limitations of this study

- A major strength of this study was the large sample, which was representative of adults living in England.
- Another strength was the broad range of smoking outcomes assessed, offering a detailed view of smoking behaviour among people living in social housing compared with those living in other housing types.
- The main limitation was that all outcomes were self-reported, introducing scope for bias.

For peer review only

Introduction

Tobacco smoking is one of the leading drivers of health inequalities in England (1). Higher smoking prevalence is associated with almost every indicator of socioeconomic disadvantage (2) and progress to reduce smoking prevalence has historically been slower among disadvantaged groups (3,4). Understanding and alleviating this inequality is a priority for public health research and policy.

Housing tenure is an indicator of socioeconomic position that is particularly strongly linked with smoking (5). A large survey in England in 2015-17 revealed 34% of adults living in social housing were smokers, compared with 15% of people living in other housing types (e.g. home owners or private renters) (6). Strikingly, smokers living in social housing were no less motivated to quit, but were only around half as likely to be successful when they tried (6). This report prompted calls for targeted action to address this disparity (7). The UK Government's 2017 tobacco control plan for England committed to eliminating inequalities and reducing smoking prevalence in groups with the highest rates (8). More recently, the Government committed to 'levelling up' disparities in health outcomes, incomes, and educational opportunities (9). What, if any, subsequent progress has been made in tackling smoking in social housing is unclear.

Using data from a nationally-representative survey of more than 100,000 adults in England between 2015 and 2020, this study aimed to provide an update on smoking in social housing in England and evaluate progress toward reducing disparities in smoking prevalence among residents of social housing compared with other housing types.

Method

Design and population

This was a cross-sectional national survey of a representative sample of adults in England. Data on housing tenure, smoking, and smoking cessation were collected in the Smoking Toolkit Study between January 2015 and February 2020 [23]. Data on housing tenure have not been collected since the Covid-19 pandemic required data collection to move from face-to-face to telephone interviews in March 2020, so these are the most up-to-date data available.

The Smoking Toolkit Study uses a hybrid of random probability and simple quota sampling to select a new sample of approximately 1,700 adults aged ≥16 years each month. Full details of the study's

For peer review only - http://bmjopen.bmj.com/site/about/guidelines.xhtml

methods are available elsewhere, and comparisons with national data and cigarette sales indicate that key variables such as sociodemographic characteristics and smoking prevalence are nationally representative (10,11).

Patient and public involvement

The wider toolkit study has been discussed with a diverse patient and public involvement (PPI) group, and the authors regularly attend and present at meetings at which patients and public are included. Interaction and discussion at these events help to shape the broad research priorities and questions. There is also a mechanism for generalised input from the wider public: each month interviewers seek feedback on the questions from all 1,700 respondents, who are representative of the English population. This feedback is limited, and usually simply relates to understanding of questions and item options. No patients or members of the public were involved in setting the research questions or the outcome measures, nor were they involved in the design and implementation of this specific study. There are no plans to involve patients in dissemination.

Measures

Housing tenure was categorised as 'social housing' (homes belonging to a housing association or rented from local authority; coded 1) vs. 'other housing' (homes bought on a mortgage, owned outright, rented from private landlord, or other; coded 0).

The smoking outcomes examined were:

(i) *among all adults*: cigarette smoking prevalence;

(ii) *among current smokers*: mean cigarettes per day (CPD) and percentage who smoke within 30 minutes of waking (as markers of cigarette dependence), high motivation to stop ('really want and plan to stop within 3 months' (12)), and regular exposure to smoking by others;

(iii) *among past-year smokers*: percentage with a past-year quit attempt; and

(iv) *among smokers with quit attempts in the past year*: percentage not currently smoking, and who used cessation support (behavioural, nicotine replacement therapy (NRT) over the counter (OTC), electronic cigarettes (e-cigarettes), or prescription medication).

Covariates were sex, age, occupational social grade (assessed using the National Readership Survey classification (13)), government office region, and survey year.

Statistical analysis

For peer review only - http://bmjopen.bmj.com/site/about/guidelines.xhtml

Data were analysed using SPSS V.27. Variables were weighted using rim (marginal) weighting to match an English population profile relevant to the time each monthly survey was conducted on the dimensions of age, social grade, region, housing tenure, ethnicity and working status within sex derived from English census data, ONS mid-year estimates and other random probability surveys (10). Missing data were removed on a per-analysis basis for each outcome.

We used linear regression (continuous outcomes) and logistic regression (binary outcomes) models to analyse associations between housing tenure (social housing vs. other housing) and smoking outcomes, with and without adjustment for covariates. To test whether the effectiveness of use of evidence-based support for cessation differed by housing tenure, accounting for differences in dependence, we used logistic regression to test the interaction between housing tenure and use of evidence-based support, adjusting for covariates and measures of dependence (cigarettes per day and smoking within 30 minutes of waking).

To examine differences in smoking prevalence trends by housing tenure over the study period, we graphically displayed annual data and reran the adjusted logistic regression model for smoking prevalence adding the interaction term between housing tenure and survey year (modelled as a continuous variable). We then ran stratified analyses in which the association between smoking prevalence and survey year was tested separately for each housing type (social vs. other) to provide more information as to the nature of the difference between groups.

Results

A total of 105,562 adults aged ≥16 years responded to the Smoking Toolkit Study survey between January 2015 and February 2020. Sample characteristics are shown in Table 1.

For peer review only - http://bmjopen.bmj.com/site/about/guidelines.xhtml

Table 1. Sample characteristics

	Total (n=105,562)		Social housing residents (n=13,862)		Other housing residents (n=91,700)	
	n	%	n	%	n	%
Female	53,830	51.0	8,105	58.5	45,725	49.9
Age (years)						
16-24	14,867	14.1	2,101	15.2	12,766	13.9
25-34	17,744	16.8	2,783	20.1	14,960	16.3
35-44	17,068	16.2	2,300	16.6	14,768	16.1
45-54	18,190	17.2	2,312	16.7	15,878	17.3
55-64	14,924	14.1	1,739	12.5	13,185	14.4
65+	22,769	21.6	2,626	18.9	20,142	22.0
Social grade*						
AB	28,649	27.1	719	5.2	27,930	30.5
C1	29,420	27.9	2,227	16.1	27,193	29.7
C2	22,389	21.2	3,351	24.2	19,038	20.8
D	15,742	14.9	3,802	27.4	11,940	13.0
E	9,362	8.9	3,764	27.2	5,598	6.1
Government office region						
North East	5,181	4.9	887	6.4	4,294	4.7
North West	13,915	13.2	1,642	11.8	12,273	13.4
Yorkshire and the Humber	10,553	10.0	1,193	8.6	9,360	10.2
East Midlands	9,164	8.7	1,224	8.8	7,940	8.7
West Midlands	10,850	10.3	1,413	10.2	9,437	10.3
East of England	11,851	11.2	1,752	12.6	10,098	11.0
London	16,110	15.3	2,782	20.1	13,328	14.5
South East	17,148	16.2	1,733	12.5	15,415	16.8
South West	10,788	10.2	1,235	8.9	9,553	10.4

*AB = managerial, administrative, and professional; C1 = supervisory, clerical and junior managerial, administrative and professional; C2 = skilled manual workers; D semi-skilled and unskilled manual workers; E = State pensioners, casual and lowest grade workers, unemployed with state benefits only.

For peer review only

Associations between housing tenure and smoking outcomes are shown in Table 2. After adjustment for sex, age, social grade, region, and survey year, adults living in social housing had more than double the odds of being a smoker compared with those living in other housing types. Current smokers living in social housing smoked on average one more cigarette per day and had 50% higher odds of smoking their first cigarette of the day within 30 minutes of waking, indicating significantly higher levels of addiction. Their level of motivation to stop smoking did not differ significantly from those living in other housing types, nor did the odds of reporting regular exposure to smoking by others. Smokers living in social housing had 16%

For peer review only - http://bmjopen.bmj.com/site/about/guidelines.xhtml

higher odds of having made a serious attempt to quit in the past year than those living in other housing types. Among smokers who had tried to quit in the past year, those living in social housing had 22% higher odds of using evidence-based cessation support (specifically, e-cigarettes or prescription medication) but 37% lower odds of remaining abstinent. This does not mean evidence-based cessation support was less effective for smokers living in social housing: after adjustment for level of dependence, the association between use of evidence-based support and cessation did not differ significantly by housing tenure (interaction OR_{adj} 0.93, 95% CI 0.64-1.34, p=0.684).

For peer review only

Table 2. Smoking and cessation behaviour in social housing compared to other housing, January 2015 to February 2020 (n=105,616)

	Social housing	Other housing	Unadjusted			Adjusted**		
			OR/B*	95% CI	*p*	OR/B	95% CI	*p*
All adults	n=13,862	n=91,700						
% Cigarette smokers	33.5	14.8	2.91	2.80-3.03	<0.001	2.17	2.08-2.27	<0.001
Current cigarette smokers	n=4,637	n=13,525						
Mean cigarettes per day	12.2	10.5	1.72	1.45-1.99	<0.001	0.99	0.71-1.27	<0.001
% First smoke within 30 min of waking	57.4	42.6	1.82	1.70-1.94	<0.001	1.50	1.39-1.61	<0.001
% High motivation to stop	14.7	15.0	0.97	0.89-1.07	0.575	1.06	0.96-1.17	0.284
% Regular exposure to smoking by others	68.4	68.6	0.99	0.92-1.06	0.778	1.01	0.94-1.10	0.749
Past-year smokers	n=4,923	n=15,054						
% Past year quit attempt	32.4	30.9	1.07	1.00-1.15	0.054	1.16	1.07-1.25	<0.001
Past year quit attempt	n=1,551	n=4,530						
% Not currently smoking	11.6	18.9	0.56	0.47-0.67	<0.001	0.63	0.52-0.76	<0.001
% Used any cessation support***	59.0	54.4	1.20	1.07-1.35	0.002	1.22	1.07-1.39	0.003
% Used behavioural support	2.8	2.2	1.25	0.87-1.80	0.229	1.20	0.80-1.80	0.377
% Used NRT OTC	13.4	13.0	1.04	0.88-1.23	0.671	0.88	0.73-1.07	0.189
% Used e-cigarettes	33.9	32.1	1.08	0.96-1.23	0.196	1.19	1.04-1.36	0.012
% Used prescription medication	9.0	7.1	1.28	1.04-1.58	0.020	1.33	1.05-1.68	0.017

*B can be interpreted as the mean (unadjusted/adjusted, as relevant) difference between the social housing and other housing groups. **OR/B adjusted for sex, age, social grade, government office region, and survey year. ***Any cessation support includes behavioural support, nicotine replacement therapy (NRT) bought over-the-counter (OTC), e-cigarettes, and prescription medication.

Number of missing cases per variable: % cigarette smokers n=51 (0.0%); mean cigarettes per day n=325 (1.8%); % first smoke within 30 min of waking n=81 (0.4%); % high motivation to stop n=33 (0.2%); % regular exposure to smoking by others n=0 (0.0%); % past year quit attempt n=556 (2.8%); % not currently smoking n=0 (0.0%); % used cessation support n=0 (0.0%).

For peer review only - http://bmjopen.bmj.com/site/about/guidelines.xhtml

Figure 1 shows annual smoking prevalence estimates over the study period. There was a significant interaction between housing tenure and survey year on smoking prevalence (OR_{adj} 1.03, 95% CI 1.01-1.06, *p*=0.020). Stratified analyses showed that there was a significant linear decline in smoking prevalence between 2015 and 2020 among adults living in other housing types (OR_{adj} 0.95, 95% CI 0.94-0.96, *p*<0.001), with prevalence falling by 24% (from 16.0% in 2015 to 12.1% in 2020). However, the decline among adults living in social housing over the same period was not statistically significant (OR_{adj} 0.98, 95% CI 0.96-1.01, *p*=0.120), falling by just 7% (from 35.3% in 2015 to 32.7% in 2020).

For peer review only

Discussion

This study extends the existing evidence base on smoking in social housing in England. Results showed adults who live in social housing remain more likely to smoke, and the general decline in smoking prevalence over recent years has stalled in this high-risk group compared with adults living in other housing types, indicating worsening inequalities in smoking on this measure. While smokers living in social housing are more addicted than those living in other housing, they are equally motivated to quit, more likely to make a quit attempt, and more likely to use support. Yet they are less likely to be successful in stopping.

The results are consistent with those of a previous analysis that included data from 2015-17 (6), suggesting there has been little change in smoking inequalities between adults who live in social versus other types of housing over recent years. The only notable difference was that in this analysis, use of prescription medication as a cessation aid was significantly higher among smokers living in social housing than other housing types when it had not been previously. This could be explained by a smaller reduction in use of prescription medication from the original to current analysis among smokers living in social housing (from 9.3% to 9.0%) than those living in other housing types (from 8.2% to 7.1%). It is encouraging that smokers in social housing were more likely to access evidence-based support, which can substantially increase their chances of quitting successfully, because their higher levels of dependence and various social and environmental barriers make it more difficult for them to successfully stop smoking. However, with four in ten quitters not using any form of evidence-based support, there remains room for improvement in helping smokers in social housing (and other housing tenures) to access effective support and translate more quit attempts into long-term cessation.

For peer review only - http://bmjopen.bmj.com/site/about/guidelines.xhtml

Without targeted action, smoking-related disparities are likely to have significant implications for the health of people and their families living in social housing. The adverse effects of smoking on health and life expectancy are well established, and the transmission to the next generation (14), but much of the harm caused by smoking can be reversed by quitting (15,16). This offers huge policy potential to ‘level up’ and reduce the damage smoking causes. Various approaches have been suggested to better support smokers in social housing, including ways in which social landlords can maximise their opportunity to improve tenants’ wellbeing (7). Most recently, the All Party Parliamentary Group on Smoking and Health recommended an at-scale intervention to provide free e-cigarettes and behavioural support to smokers in social housing (17) based on a successful pilot in Salford in the North of England (18).

A major strength of this study was the large, representative sample. The main limitation was that all outcomes were self-reported, introducing scope for bias. Measurement of quit attempts and use of support relied on recall of the past year and quit success was not biochemically verified. While the latter would be a significant limitation in randomised trials (because smokers who receive active treatment may feel social pressure to claim abstinence) social pressure and the associated rate of misreporting is low in population surveys (19). Moreover, we would not expect the extent of misreporting to differ by housing tenure meaning our results are unlikely to materially be affected.

In conclusion, there remain stark inequalities in smoking and quitting behaviour by housing tenure in England, with declines in prevalence stalling between 2015 and 2020 despite progress in the rest of the population. In the absence of targeted interventions to boost quitting among social housing residents, inequalities in health are likely to worsen. In the context of the UK Government’s commitment to levelling up, tackling smoking in social housing should be an urgent priority.

For peer review only

For peer review only - http://bmjopen.bmj.com/site/about/guidelines.xhtml

Figure legends

Figure 1. Annual smoking prevalence among adults in England living in social housing compared with other housing tenures, January 2015 through February 2020. Shaded bands indicate 95% confidence intervals. Bases (weighted n): social housing 2015 n=2849, 2016 n=2910, 2017 n=2717, 2018 n=2579, 2019 n=2420, 2020 n=373; other housing 2015 n=17132, 2016 n=17520, 2017 n=17662, 2018 n=18106, 2019 n=18215, 2020 n=3029. *Note: Data for 2020 are from January and February only.

For peer review only

For peer review only - http://bmjopen.bmj.com/site/about/guidelines.xhtml

Declarations

Competing interests

JB has received unrestricted research funding from Pfizer, who manufacture smoking cessation medications. All authors declare no financial links with tobacco companies or e-cigarette manufacturers or their representatives.

Funding

This work was supported by Cancer Research UK (C1417/A22962).

Author contributions

All authors conceived and designed the study. SJ analysed the data and wrote the first draft. All authors provided critical revisions.

Ethical approval

Ethical approval for the STS was granted by the UCL Ethics Committee (ID 0498/001). The data are not collected by UCL and are anonymised when received by UCL.

Data sharing

Data are available on request from the corresponding author.

For peer review only

For peer review only - http://bmjopen.bmj.com/site/about/guidelines.xhtml

References

1. Institute of Health Equity. Fair Society Healthy Lives (The Marmot Review) [Internet]. 2010 [cited 2018 Oct 23]. Available from: http://www.instituteofhealthequity.org/resources-reports/fair-society-healthy-lives-the-marmot-review

2. Hiscock R, Bauld L, Amos A, Fidler JA, Munafò M. Socioeconomic status and smoking: a review. Ann N Y Acad Sci. 2012 Feb;1248:107–23.

3. Office for National Statistics. Adult smoking habits in the UK: 2019 [Internet]. 2020. Available from: https://www.ons.gov.uk/peoplepopulationandcommunity/healthandsocialcare/healthandlifeexpectancies/bulletins/adultsmokinghabitsingreatbritain/2019#the-proportion-who-are-current-smokers-in-the-uk-its-consistent-countries-and-local-areas-2011-to-2019

4. Jarvis MJ, Wardle J. Social patterning of individual health behaviours: the case of cigarette smoking. Soc Determinants Health. 1999;2:224–37.

5. Beard E, Brown J, Jackson SE, West R, Kock L, Boniface S, et al. Independent associations between different measures of socioeconomic position and smoking status: A cross-sectional study of adults in England. Nicotine Tob Res [Internet]. 2020 [cited 2020 Mar 19]; Available from: https://academic.oup.com/ntr/advance-article/doi/10.1093/ntr/ntaa030/5728574

6. Jackson SE, Smith C, Cheeseman H, West R, Brown J. Finding smoking hot-spots: a cross-sectional survey of smoking patterns by housing tenure in England. Addiction. 2019;114(5):889–95.

7. Action on Smoking and Health. Smoking in the home: New solutions for a Smokefree Generation [Internet]. 2018 [cited 2019 Sep 4]. Available from: http://ash.org.uk/wp-content/uploads/2018/11/FINAL-2018-Smokefree-Housing-report-web.pdf

8. Department of Health. Towards a Smokefree Generation: A Tobacco Control Plan for England [Internet]. London: Department of Health; 2017 Jul [cited 2018 Sep 13]. Available from: https://assets.publishing.service.gov.uk/government/uploads/system/uploads/attachment_data/file/630217/Towards_a_Smoke_free_Generation_-_A_Tobacco_Control_Plan_for_England_2017-2022__2_.pdf

9. HM Treasury. Build Back Better: our plan for growth [Internet]. 2021 [cited 2021 Aug 2]. Available from: https://assets.publishing.service.gov.uk/government/uploads/system/uploads/attachment_data/file/968403/PfG_Final_Web_Accessible_Version.pdf

10. Fidler JA, Shahab L, West O, Jarvis MJ, McEwen A, Stapleton JA, et al. “The smoking toolkit study”: a national study of smoking and smoking cessation in England. BMC Public Health. 2011 Jun 18;11:479.

11. Jackson SE, Beard E, Kujawski B, Sunyer E, Michie S, Shahab L, et al. Comparison of Trends in Self-reported Cigarette Consumption and Sales in England, 2011 to 2018. JAMA Netw Open. 2019 Aug 2;2(8):e1910161.

12. Kotz D, Brown J, West R. Predictive validity of the Motivation To Stop Scale (MTSS): A single-item measure of motivation to stop smoking. Drug Alcohol Depend. 2013 Feb 1;128(1):15–9.

For peer review only - http://bmjopen.bmj.com/site/about/guidelines.xhtml

13. National Readership Survey. Social grade - definitions and discriminatory power [Internet]. 2007 [cited 2012 Oct 1]. Available from: http:// www.nrs.co.uk/lifestyle.html

14. Leonardi-Bee J, Jere ML, Britton J. Exposure to parental and sibling smoking and the risk of smoking uptake in childhood and adolescence: a systematic review and meta-analysis. Thorax. 2011 Oct 1;66(10):847–55.

15. Doll R, Peto R, Boreham J, Sutherland I. Mortality in relation to smoking: 50 years' observations on male British doctors. BMJ. 2004 Jun 24;328(7455):1519.

16. Pirie K, Peto R, Reeves GK, Green J, Beral V. The 21st century hazards of smoking and benefits of stopping: a prospective study of one million women in the UK. The Lancet. 2013 Jan 12;381(9861):133–41.

17. APPG on Smoking and Health. Delivering a Smokefree 2030: The All Party Parliamentary Group on Smoking and Health recommendations for the Tobacco Control Plan 2021 [Internet]. London; 2021 [cited 2021 Dec 13]. Available from: https://ash.org.uk/about-ash/all-party-parliamentary-group-on-smoking-health/inquiries-reports/deliveringasf2030appgtcp2021/

18. Greater Manchester Combined Authority, NHS in Greater Manchester, University of Salford, Salford City Council. E-cigarette swap to stop evaluation summary [Internet]. Manchester, UK; 2020 [cited 2021 Dec 13]. Available from: https://www.gmhsc.org.uk/wp-content/uploads/2020/08/E-cig-Eval-Summary19_FINAL.pdf

19. West R, Zatonski W, Przewozniak K, Jarvis MJ. Can we trust national smoking prevalence figures? Discrepancies between biochemically assessed and self-reported smoking rates in three countries. Cancer Epidemiol Biomark Prev Publ Am Assoc Cancer Res Cosponsored Am Soc Prev Oncol. 2007 Apr;16(4):820–2.

For peer review only

For peer review only - http://bmjopen.bmj.com/site/about/guidelines.xhtml

For peer review only

For peer review only - http://bmjopen.bmj.com/site/about/guidelines.xhtml

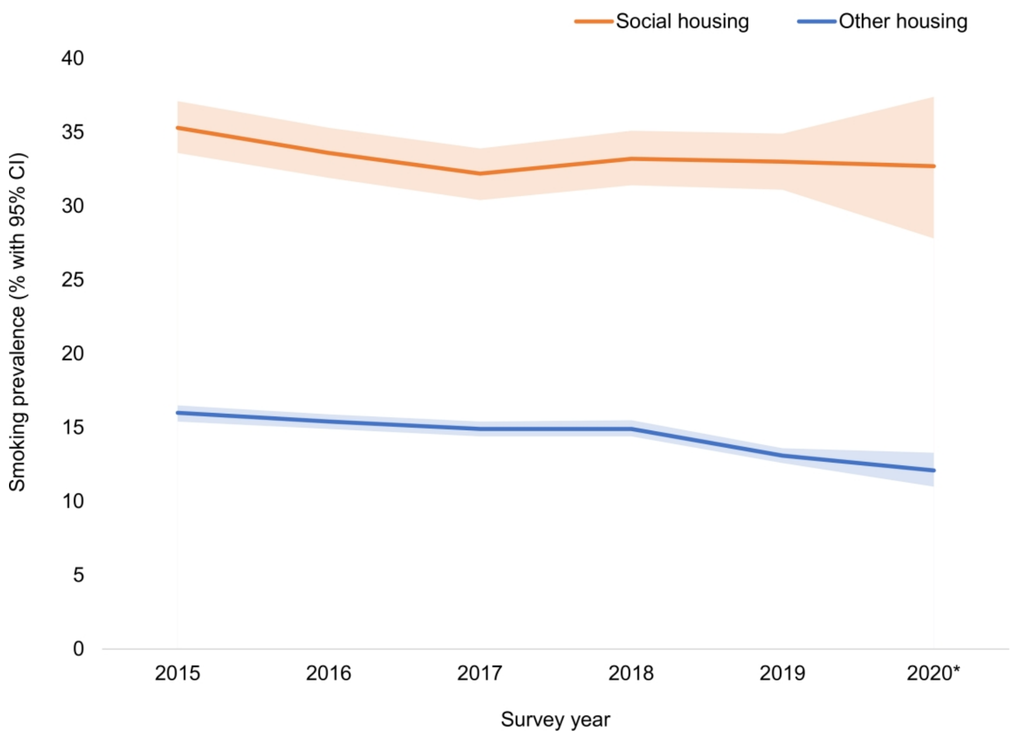

Figure 1. Annual smoking prevalence among adults in England living in social housing compared with other housing tenures, January 2015 through February 2020. Shaded bands indicate 95% confidence intervals. Bases (weighted n): social housing 2015 n=2849, 2016 n=2910, 2017 n=2717, 2018 n=2579, 2019 n=2420, 2020 n=373; other housing 2015 n=17132, 2016 n=17520, 2017 n=17662, 2018 n=18106, 2019 n=18215, 2020 n=3029. *Note: Data for 2020 are from January and February only.

188x135mm (300 x 300 DPI)

STROBE Statement—Checklist of items that should be included in reports of ***cross-sectional studies***

	Item No	Recommendation	Page No
**Title and abstract**	1	(*a*) Indicate the study's design with a commonly used term in the title or the abstract	1
		(*b*) Provide in the abstract an informative and balanced summary of what was done and what was found	2
**Introduction**			
Background/rationale	2	Explain the scientific background and rationale for the investigation being reported	4
Objectives	3	State specific objectives, including any prespecified hypotheses	4
**Methods**			
Study design	4	Present key elements of study design early in the paper	4
Setting	5	Describe the setting, locations, and relevant dates, including periods of recruitment, exposure, follow-up, and data collection	4
Participants	6	(*a*) Give the eligibility criteria, and the sources and methods of selection of participants	4
Variables	7	Clearly define all outcomes, exposures, predictors, potential confounders, and effect modifiers. Give diagnostic criteria, if applicable	5
Data sources/ measurement	8*	For each variable of interest, give sources of data and details of methods of assessment (measurement). Describe comparability of assessment methods if there is more than one group	5
Bias	9	Describe any efforts to address potential sources of bias	6
Study size	10	Explain how the study size was arrived at	6
Quantitative variables	11	Explain how quantitative variables were handled in the analyses. If applicable, describe which groupings were chosen and why	5
Statistical methods	12	(*a*) Describe all statistical methods, including those used to control for confounding	6
		(*b*) Describe any methods used to examine subgroups and interactions	6
		(*c*) Explain how missing data were addressed	6
		(*d*) If applicable, describe analytical methods taking account of sampling strategy	6
		(*e*) Describe any sensitivity analyses	6
**Results**			
Participants	13*	(a) Report numbers of individuals at each stage of study—eg numbers potentially eligible, examined for eligibility, confirmed eligible, included in the study, completing follow-up, and analysed	6
		(b) Give reasons for non-participation at each stage	n/a
		(c) Consider use of a flow diagram	n/a
Descriptive data	14*	(a) Give characteristics of study participants (eg demographic, clinical, social) and information on exposures and potential confounders	7
		(b) Indicate number of participants with missing data for each variable of interest	9
Outcome data	15*	Report numbers of outcome events or summary measures	9
Main results	16	(*a*) Give unadjusted estimates and, if applicable, confounder-adjusted estimates and their precision (eg, 95% confidence interval). Make clear which confounders were adjusted for and why they were included	9

For peer review only

		(*b*) Report category boundaries when continuous variables were categorized	n/a
		(*c*) If relevant, consider translating estimates of relative risk into absolute risk for a meaningful time period	n/a
Other analyses	17	Report other analyses done—eg analyses of subgroups and interactions, and sensitivity analyses	10
**Discussion**			
Key results	18	Summarise key results with reference to study objectives	11
Limitations	19	Discuss limitations of the study, taking into account sources of potential bias or imprecision. Discuss both direction and magnitude of any potential bias	12
Interpretation	20	Give a cautious overall interpretation of results considering objectives, limitations, multiplicity of analyses, results from similar studies, and other relevant evidence	11-12
Generalisability	21	Discuss the generalisability (external validity) of the study results	12
**Other information**			
Funding	22	Give the source of funding and the role of the funders for the present study and, if applicable, for the original study on which the present article is based	15

*Give information separately for exposed and unexposed groups.

Note: An Explanation and Elaboration article discusses each checklist item and gives methodological background and published examples of transparent reporting. The STROBE checklist is best used in conjunction with this article (freely available on the Web sites of PLoS Medicine at http://www.plosmedicine.org/, Annals of Internal Medicine at http://www.annals.org/, and Epidemiology at http://www.epidem.com/). Information on the STROBE Initiative is available at www.strobe-statement.org.

For peer review only

BMJ Open

Smoking in social housing among adults in England, 2015-2020: a nationally representative survey

Journal:	*BMJ Open*
Manuscript ID	bmjopen-2022-061013.R1
Article Type:	Original research
Date Submitted by the Author:	29-Jun-2022
Complete List of Authors:	Jackson, Sarah ; UCL, Behavioural Science and Health<br>Cheeseman, Hazel; Action on Smoking and Health<br>Arnott, Deborah; Action on Smoking and Health<br>Titmarsh, Robbie; Action on Smoking and Health<br>Brown, Jamie; UCL, Behavioural Science and Health
**Primary Subject Heading**:	Addiction
Secondary Subject Heading:	Epidemiology
Keywords:	EPIDEMIOLOGY, PUBLIC HEALTH, GENERAL MEDICINE (see Internal Medicine)

SCHOLARONE™
Manuscripts

I, the Submitting Author has the right to grant and does grant on behalf of all authors of the Work (as defined in the below author licence), an exclusive licence and/or a non-exclusive licence for contributions from authors who are: i) UK Crown employees; ii) where BMJ has agreed a CC-BY licence shall apply, and/or iii) in accordance with the terms applicable for US Federal Government officers or employees acting as part of their official duties; on a worldwide, perpetual, irrevocable, royalty-free basis to BMJ Publishing Group Ltd ("BMJ") its licensees and where the relevant Journal is co-owned by BMJ to the co-owners of the Journal, to publish the Work in this journal and any other BMJ products and to exploit all rights, as set out in our licence.

The Submitting Author accepts and understands that any supply made under these terms is made by BMJ to the Submitting Author unless you are acting as an employee on behalf of your employer or a postgraduate student of an affiliated institution which is paying any applicable article publishing charge ("APC") for Open Access articles. Where the Submitting Author wishes to make the Work available on an Open Access basis (and intends to pay the relevant APC), the terms of reuse of such Open Access shall be governed by a Creative Commons licence – details of these licences and which Creative Commons licence will apply to this Work are set out in our licence referred to above.

Other than as permitted in any relevant BMJ Author's Self Archiving Policies, I confirm this Work has not been accepted for publication elsewhere, is not being considered for publication elsewhere and does not duplicate material already published. I confirm all authors consent to publication of this Work and authorise the granting of this licence.

For peer review only

Smoking in social housing among adults in England, 2015-2020: a nationally representative survey

Sarah E. Jackson[1,2], Hazel Cheeseman[3], Deborah Arnott[3], Robbie Titmarsh[3], Jamie Brown[1,2]

[1] Department of Behavioural Science and Health, University College London, London, UK

[2] SPECTRUM Consortium, UK

[3] Action on Smoking and Health, London, UK

Corresponding author: Dr Sarah Jackson, Department of Behavioural Science and Health, University College London, 1-19 Torrington Place, London WC1E 7HB, UK. s.e.jackson@ucl.ac.uk +44 (0)207 679 8312

Word count: 3046

Number of tables: 2

Number of figures: 1

For peer review only - http://bmjopen.bmj.com/site/about/guidelines.xhtml

Abstract

Objectives: To analyse associations between living in social housing and smoking in England and evaluate progress toward reducing disparities in smoking prevalence among residents of social housing compared with other housing types.

Design: Cross-sectional analysis of nationally-representative data collected between January 2015 and February 2020.

Setting: England.

Participants: 105,562 adults (≥16y).

Primary and secondary outcome measures: Linear and logistic regression were used to analyse associations between living in social housing (vs. other housing types) and smoking status, cigarettes per day, time to first cigarette, exposure to others' smoking, motivation to stop smoking, quit attempts, and use of cessation support. Analyses adjusted for sex, age, social grade, region, and year.

Results: Adults living in social housing had twice the odds of being a smoker (OR_{adj}=2.17, 95%CI 2.08-2.27), and the decline in smoking prevalence between 2015 and 2020 was less pronounced in this high-risk group (-7%; OR_{adj}=0.98, 95%CI 0.96-1.01) than among adults living in other housing types (-24%; OR_{adj}=0.95, 95%CI 0.94-0.96; housing tenure*survey year interaction *p*=0.020). Smokers living in social housing were more addicted than those in other housing (smoking within 30 minutes of waking: OR_{adj}=1.50, 95%CI 1.39-1.61), but were no less motivated to stop smoking (OR_{adj}=1.06, 95%CI 0.96-1.17) and had higher odds of having made a serious attempt to quit in the past year (OR_{adj}=1.16, 95%CI 1.07-1.25). Among smokers who had tried to quit, those living in social housing had higher odds of using evidence-based cessation support (OR_{adj}=1.22, 95%CI 1.07-1.39) but lower odds of remaining abstinent (OR_{adj}=0.63, 95%CI 0.52-0.76).

Conclusions: There remain stark inequalities in smoking and quitting behaviour by housing tenure in England, with declines in prevalence stalling between 2015 and 2020 despite progress in the rest of the population. In the absence of targeted interventions to boost quitting among social housing residents, inequalities in health are likely to worsen.

Key words: smoking; social housing; housing tenure; inequalities

For peer review only - http://bmjopen.bmj.com/site/about/guidelines.xhtml

Strengths and limitations of this study

- A major strength of this study was the large sample, which was representative of adults living in England.
- Another strength was the broad range of smoking outcomes assessed, offering a detailed view of smoking behaviour among people living in social housing compared with those living in other housing types.
- The main limitation was that all outcomes were self-reported, introducing scope for bias.

For peer review only

Introduction

Tobacco smoking is one of the leading drivers of health inequalities in England (1). Higher smoking prevalence is associated with almost every indicator of socioeconomic disadvantage (2) and progress to reduce smoking prevalence has historically been slower among disadvantaged groups (3,4). Understanding and alleviating this inequality is a priority for public health research and policy.

Housing tenure is an indicator of socioeconomic position that is particularly strongly linked with smoking (5). In particular, social housing has been identified as a potential smoking 'hot spot' (6). In England, social housing is let at lower rents on a secure, long-term basis to those who cannot afford to rent or buy a home on the open market, with priority given to those who have the greatest need. Accommodation is funded and regulated by the government and owned and managed by local authorities (local councils made up of publicly elected councillors) or housing associations (independent, not-for-profit organisations). A large survey in England in 2015-17 revealed 34% of adults living in social housing were smokers, compared with 15% of people living in other housing types (e.g. home owners or private renters) (6). Strikingly, smokers living in social housing were no less motivated to quit, but were only around half as likely to be successful when they tried (6). This report prompted calls for targeted action to address this disparity (7). The UK Government's 2017 tobacco control plan for England committed to eliminating inequalities and reducing smoking prevalence in groups with the highest rates (8). More recently, the Government committed to 'levelling up' disparities in health outcomes, incomes, and educational opportunities (9). What, if any, subsequent progress has been made in tackling smoking in social housing is unclear.

Using data from a nationally-representative survey of more than 100,000 adults in England between 2015 and 2020, this study aimed to provide an update on smoking in social housing in England and evaluate progress toward reducing disparities in smoking prevalence among residents of social housing compared with other housing types.

Method

Ethical approval

The data are collected by Ipsos Mori on behalf of UCL and are anonymised before being received by UCL. Approval for the study was granted by UCL Ethics Committee (ID 0498/001). Explicit verbal

For peer review only - http://bmjopen.bmj.com/site/about/guidelines.xhtml

agreement and willingness to answer questions voluntarily is recorded electronically by Ipsos Mori. Participants are also given a printed information sheet.

Design and population

Data were drawn from the Smoking Toolkit Study, a monthly cross-sectional survey representative of adults in England designed to provide insights into population-wide influences on smoking and cessation by monitoring trends on a range of variables relating to smoking (10).

The Smoking Toolkit Study uses a hybrid of random probability and simple quota sampling to select a new sample of approximately 1,700 adults aged ≥16 years in England each month. To recruit each monthly sample, England is split into more than 170,000 output areas (consisting of approximately 300 households each). These output areas are stratified by ACORN characteristics (an established geo-demographic analysis of the population; http://www.caci.co.uk/acorn/ and geographic region then randomly selected to be included in an interviewer's list. Interviewers travel to the selected areas and perform computer assisted interviews with one participant aged over 16 per household until quotas based upon factors influencing the probability of being at home (working status, age, and gender) are fulfilled. Participants complete a face-to-face computer-assisted survey with a trained interviewer. Comparisons with national data and cigarette sales indicate that key variables such as sociodemographic characteristics and smoking prevalence are nationally representative (10,11).

Data on housing tenure, smoking, and smoking cessation were collected between January 2015 and February 2020, so our analyses focus on participants recruited during this period. Data on housing tenure have not been collected since the Covid-19 pandemic required data collection to move from face-to-face to telephone interviews in March 2020, so these are the most up-to-date data available.

Patient and public involvement

The wider toolkit study has been discussed with a diverse patient and public involvement (PPI) group, and the authors regularly attend and present at meetings at which patients and public are included. Interaction and discussion at these events help to shape the broad research priorities and questions. There is also a mechanism for generalised input from the wider public: each month interviewers seek feedback on the questions from all 1,700 respondents, who are representative of the English population. This feedback is limited, and usually simply relates to understanding of questions and item options. No patients or members of the public were involved in setting the

For peer review only - http://bmjopen.bmj.com/site/about/guidelines.xhtml

research questions or the outcome measures, nor were they involved in the design and implementation of this specific study. There are no plans to involve patients in dissemination.

Measures

Housing tenure was categorised as ‘social housing’ (homes belonging to a housing association or rented from local authority; coded 1) vs. ‘other housing’ (homes bought on a mortgage, owned outright, rented from private landlord, or other; coded 0).

The smoking outcomes examined were:

(i) *among all adults*: cigarette smoking prevalence;

(ii) *among current smokers*: mean cigarettes per day (CPD) and percentage who smoke within 30 minutes of waking (as markers of cigarette dependence), high motivation to stop (‘really want and plan to stop within 3 months’ (12)), and regular exposure to smoking by others;

(iii) *among past-year smokers*: percentage with a past-year quit attempt; and

(iv) *among smokers with quit attempts in the past year*: percentage not currently smoking, and who used cessation support (behavioural, nicotine replacement therapy (NRT) over the counter (OTC), electronic cigarettes (e-cigarettes), or prescription medication).

Covariates were sex, age, occupational social grade (assessed using the National Readership Survey classification (13)), government office region, and survey year.

Statistical analysis

Data were analysed using SPSS V.27. Variables were weighted using rim (marginal) weighting to match an English population profile relevant to the time each monthly survey was conducted on the dimensions of age, social grade, region, housing tenure, ethnicity and working status within sex derived from English census data, ONS mid-year estimates and other random probability surveys (10). Missing data were removed on a per-analysis basis for each outcome.

We used linear regression (continuous outcomes) and logistic regression (binary outcomes) models to analyse associations between housing tenure (social housing vs. other housing) and smoking outcomes, with and without adjustment for covariates. To test whether the effectiveness of use of evidence-based support for cessation differed by housing tenure, accounting for differences in dependence, we used logistic regression to test the interaction between housing tenure and use of evidence-based support, adjusting for covariates and measures of dependence (cigarettes per day and smoking within 30 minutes of waking).

For peer review only - http://bmjopen.bmj.com/site/about/guidelines.xhtml

Following peer review, we reran these analyses using log-binomial regression as an alternative to logistic regression, to explore any differences in results. We also repeated our adjusted models with the inclusion of interactions between housing tenure and (i) age (16-34, 35-64, and ≥65 years) and (ii) sex, to test for moderation of associations by these characteristics. Each interaction was tested in a separate model. Where interactions were statistically significant, we ran stratified analyses in which the association between housing tenure and the outcome variable was tested separately for each level of the moderating variable (i.e. separately by age group or sex) to provide more information as to the nature of the differences between groups.

To examine differences in smoking prevalence trends by housing tenure over the study period, we graphically displayed annual data and reran the adjusted logistic regression model for smoking prevalence adding the interaction term between housing tenure and survey year (modelled as a continuous variable). We then ran stratified analyses in which the association between smoking prevalence and survey year was tested separately for each housing type (social vs. other) to provide more information as to the nature of the difference between groups.

Results

A total of 105,562 adults aged ≥16 years responded to the Smoking Toolkit Study survey between January 2015 and February 2020. Sample characteristics are shown in **Table 1**. A total of 13,862 participants (13.1%) were social housing residents. Those living in social housing were more likely to be female, younger, and from more disadvantaged social grades, and were more likely to live in London.

For peer review only - http://bmjopen.bmj.com/site/about/guidelines.xhtml

Table 1. Sample characteristics

	Total (n=105,562)		Social housing residents (n=13,862)		Other housing residents (n=91,700)	
	n	%	n	%	n	%
Female	53,830	51.0	8,105	58.5	45,725	49.9
Age (years)						
16-24	14,867	14.1	2,101	15.2	12,766	13.9
25-34	17,744	16.8	2,783	20.1	14,960	16.3
35-44	17,068	16.2	2,300	16.6	14,768	16.1
45-54	18,190	17.2	2,312	16.7	15,878	17.3
55-64	14,924	14.1	1,739	12.5	13,185	14.4
65+	22,769	21.6	2,626	18.9	20,142	22.0
Social grade*						
AB (most advantaged)	28,649	27.1	719	5.2	27,930	30.5
C1	29,420	27.9	2,227	16.1	27,193	29.7
C2	22,389	21.2	3,351	24.2	19,038	20.8
D	15,742	14.9	3,802	27.4	11,940	13.0
E (most disadvantaged)	9,362	8.9	3,764	27.2	5,598	6.1
Government office region						
North East	5,181	4.9	887	6.4	4,294	4.7
North West	13,915	13.2	1,642	11.8	12,273	13.4
Yorkshire and the Humber	10,553	10.0	1,193	8.6	9,360	10.2
East Midlands	9,164	8.7	1,224	8.8	7,940	8.7
West Midlands	10,850	10.3	1,413	10.2	9,437	10.3
East of England	11,851	11.2	1,752	12.6	10,098	11.0
London	16,110	15.3	2,782	20.1	13,328	14.5
South East	17,148	16.2	1,733	12.5	15,415	16.8
South West	10,788	10.2	1,235	8.9	9,553	10.4
Year of survey						
2015	19,988	18.9	2,849	20.6	17,139	18.7
2016	20,433	19.4	2,911	21.0	17,522	19.1
2017	20,395	19.3	2,726	19.7	17,669	19.3
2018	20,703	19.6	2,584	18.6	18,119	19.8
2019	20,641	19.6	2,420	17.5	18,221	19.9
2020	3,402	3.2	373	2.7	3,029	3.3

*AB = managerial, administrative, and professional; C1 = supervisory, clerical and junior managerial, administrative and professional; C2 = skilled manual workers; D semi-skilled and unskilled manual workers; E = State pensioners, casual and lowest grade workers, unemployed with state benefits only.

For peer review only - http://bmjopen.bmj.com/site/about/guidelines.xhtml

Associations between housing tenure and smoking outcomes are shown in **Table 2**. Interactions between housing tenure and age group and sex are summarised in **Supplementary Tables 1 and 2**, respectively, and stratified results are included in Table 2 for outcomes where there is evidence of interaction with age or sex.

After adjustment for sex, age, social grade, region, and survey year, adults living in social housing had more than double the odds of being a smoker compared with those living in other housing types. While this association was observed across all age groups and sexes, it was more pronounced among over 35s (vs. 16-34y) and women (vs. men).

Current smokers living in social housing smoked on average one more cigarette per day and had 50% higher odds of smoking their first cigarette of the day within 30 minutes of waking, indicating significantly higher levels of addiction. These associations were strongest among younger adults (16-34y), weaker among middle-aged adults (35-64y), and were not statistically significant in the oldest group (≥65y). Motivation to stop smoking did not differ significantly by housing tenure, nor did the odds of reporting regular exposure to smoking by others.

Smokers living in social housing had 16% higher odds of having made a serious attempt to quit in the past year than those living in other housing types. Among smokers who had tried to quit in the past year, those living in social housing had 22% higher odds of using evidence-based cessation support (specifically, e-cigarettes or prescription medication). This difference was driven by smokers in the youngest age group (16-34y), with no significant difference in use of support by housing tenure among middle-aged and older smokers. Despite greater use of support, smokers living in social housing had 37% lower odds of remaining abstinent after making a quit attempt. This does not mean evidence-based cessation support was less effective for smokers living in social housing: after adjustment for level of dependence, the association between use of evidence-based support and cessation did not differ significantly by housing tenure (interaction OR_{adj} 0.93, 95% CI 0.64-1.34, *p*=0.684; **Supplementary Table 3**).

There was little difference in the pattern of results when data were analysed using log-binomial regression (**Supplementary Table 4**), although the difference in the rate of use of cessation support became non-significant (RR_{adj} 1.09, 95% CI 0.99-1.21, *p*=0.086).

For peer review only - http://bmjopen.bmj.com/site/about/guidelines.xhtml

Table 2. Smoking and cessation behaviour in social housing compared to other housing, January 2015 to February 2020 (n=105,616), presented overall and stratified by age and sex where indicated by interactions

	Social housing	Other housing	Unadjusted			Adjusted[2]		
			OR/*B*[1]	95% CI	*p*	OR/*B*	95% CI	*p*
**All adults**[3]								
% Cigarette smokers	33.5	14.8	2.91	2.80 to 3.03	<0.001	2.17	2.08 to 2.27	<0.001
Age 18-34	35.8	20.3	2.19	2.05 to 2.34	<0.001	1.80	1.68 to 1.93	<0.001
Age 35-64	37.4	14.8	3.45	3.26 to 3.65	<0.001	2.27	2.13 to 2.42	<0.001
Age ≥65	19.7	7.1	3.20	2.87 to 3.57	<0.001	2.58	2.29 to 2.90	<0.001
Male	35.2	16.4	2.78	2.62 to 2.95	<0.001	2.02	1.90 to 2.16	<0.001
Female	32.3	13.1	3.16	2.99 to 3.33	<0.001	2.31	2.18 to 2.45	<0.001
**Current cigarette smokers**[4]								
Mean cigarettes per day	12.2	10.5	*1.72*	*1.45 to 1.99*	*<0.001*	*0.97*	*0.69 to 1.25*	*<0.001*
Age 18-34	11.0	8.8	*2.20*	*1.82 to 2.58*	*<0.001*	*1.50*	*1.11 to 1.90*	*<0.001*
Age 35-64	13.0	11.6	*1.38*	*0.98 to 1.78*	*<0.001*	*0.68*	*0.26 to 1.11*	*0.002*
Age ≥65	12.4	11.9	*0.65*	*-0.18 to 1.49*	*<0.001*	*0.32*	*-0.57 to 1.21*	*0.486*
% First smoke within 30 min of waking	57.4	42.6	1.82	1.70 to 1.94	<0.001	1.50	1.39 to 1.61	<0.001
Age 18-34	53.3	35.6	2.06	1.85 to 2.30	<0.001	1.68	1.49 to 1.90	<0.001
Age 35-64	61.8	47.9	1.76	1.60 to 1.94	<0.001	1.47	1.32 to 1.63	<0.001
Age ≥65	50.8	45.6	1.23	1.01 to 1.51	0.042	1.12	0.90 to 1.39	0.309
% High motivation to stop	14.7	15.0	0.97	0.89 to 1.07	0.575	1.06	0.96 to 1.17	0.284
% Regular exposure to smoking by others	68.4	68.6	0.99	0.92 to 1.06	0.778	1.01	0.94 to 1.10	0.749
Age 18-34	73.5	76.3	0.86	0.76 to 0.97	0.016	0.94	0.82 to 1.08	0.380
Age 35-64	67.0	65.8	1.06	0.96 to 1.17	0.279	1.05	0.94 to 1.17	0.415
Age ≥65	57.3	51.0	1.29	1.05 to 1.58	0.014	1.19	0.95 to 1.48	0.123
**Past-year smokers**[5]								
% Past-year quit attempt	32.4	30.9	1.07	1.00 to 1.15	0.054	1.16	1.07 to 1.25	<0.001

Table continued on next page.

For peer review only - http://bmjopen.bmj.com/site/about/guidelines.xhtml

Table 2. *continued.*

	Social housing	Other housing	Unadjusted			Adjusted[2]		
			OR/B[1]	95% CI	*p*	OR/B	95% CI	*p*
**Past-year quit attempt**[6]								
% Not currently smoking	11.6	18.9	0.56	0.47 to 0.67	<0.001	0.63	0.52 to 0.76	<0.001
% Used any cessation support[7]	59.0	54.4	1.20	1.07 to 1.35	0.002	1.22	1.07 to 1.39	0.003
Age 18-34	56.0	47.4	1.41	1.17 to 1.68	<0.001	1.43	1.17 to 1.74	<0.001
Age 35-64	61.9	61.2	1.03	0.87 to 1.22	0.731	1.05	0.87 to 1.27	0.591
Age ≥65	56.1	55.4	1.02	0.68 to 1.54	0.916	1.11	0.69 to 1.77	0.672
% Used behavioural support	2.8	2.2	1.25	0.87 to 1.80	0.229	1.20	0.80 to 1.80	0.377
% Used NRT OTC	13.4	13.0	1.04	0.88 to 1.23	0.671	0.88	0.73 to 1.07	0.189
% Used e-cigarettes	33.9	32.1	1.08	0.96 to 1.23	0.196	1.19	1.04 to 1.36	0.012
% Used prescription medication	9.0	7.1	1.28	1.04 to 1.58	0.020	1.33	1.05 to 1.68	0.017

[1]B can be interpreted as the mean (unadjusted/adjusted, as relevant) difference between the social housing and other housing groups. [2]OR/B adjusted for sex, age, social grade, government office region, and survey year. [3]All adults: social housing n=13,862; other housing n=91,700. [4]Current cigarette smokers: social housing n=4,637; other housing n=13,525. [5]Past-year smokers: social housing n=4,923; other housing n=15,054. [6]Past-year smokers who made a past-year quit attempt: social housing n=1,551; other housing n=4,530. [7]Any cessation support includes behavioural support, nicotine replacement therapy (NRT) bought over-the-counter (OTC), e-cigarettes, and prescription medication.

Number of missing cases per variable: % cigarette smokers n=51 (0.0%); mean cigarettes per day n=325 (1.8%); % first smoke within 30 min of waking n=81 (0.4%); % high motivation to stop n=33 (0.2%); % regular exposure to smoking by others n=0 (0.0%); % past year quit attempt n=556 (2.8%); % not currently smoking n=0 (0.0%); % used cessation support n=0 (0.0%).

Grey shading indicates results of subgroup analyses conducted when the interaction between housing tenure and age or sex (as relevant) was statistically significant.

For peer review only - http://bmjopen.bmj.com/site/about/guidelines.xhtml

Figure 1 shows annual smoking prevalence estimates over the study period. There was a significant interaction between housing tenure and survey year on smoking prevalence (OR_{adj} 1.03, 95% CI 1.01-1.06, *p*=0.020). Analyses stratified by housing tenure showed that there was a significant linear decline in smoking prevalence between 2015 and 2020 among adults living in other housing types (OR_{adj} 0.95, 95% CI 0.94-0.96, *p*<0.001), with prevalence falling by 24% (from 16.0% in 2015 to 12.1% in 2020). However, the decline among adults living in social housing over the same period was not statistically significant (OR_{adj} 0.98, 95% CI 0.96-1.01, *p*=0.120), falling by just 7% (from 35.3% in 2015 to 32.7% in 2020).

Discussion

This study extends the existing evidence base on smoking in social housing in England. Results showed adults who live in social housing remain more likely to smoke, with living in social housing particularly strongly linked to being a smoker in middle-aged and older adults and women. The general decline in smoking prevalence over recent years has stalled in this high-risk group compared with adults living in other housing types, indicating worsening inequalities in smoking on this measure. While smokers living in social housing are more addicted than those living in other housing (especially younger smokers), they are equally motivated to quit, more likely to make a quit attempt, and more likely to use support. Yet they are less likely to be successful in stopping.

The results are consistent with those of a previous analysis that included data from 2015-17 (6), suggesting there has been little change in smoking inequalities between adults who live in social versus other types of housing over recent years. A notable difference was that in this analysis, use of prescription medication as a cessation aid was significantly higher among smokers living in social housing than other housing types when it had not been previously. This could be explained by a smaller reduction in use of prescription medication from the original to current analysis among smokers living in social housing (from 9.3% to 9.0%) than those living in other housing types (from 8.2% to 7.1%). It is encouraging that younger smokers in social housing were more likely to access evidence-based support, which can substantially increase their chances of quitting successfully, because their higher levels of dependence and various social and environmental barriers make it more difficult for them to successfully stop smoking. However, with four in ten quitters not using any form of evidence-based support, there remains room for improvement in helping smokers in

For peer review only - http://bmjopen.bmj.com/site/about/guidelines.xhtml

For peer review only

social housing (and other housing tenures) to access effective support and translate more quit attempts into long-term cessation.

This analysis also provided some evidence of moderation of associations between housing tenure and smoking outcomes by age and sex. While living in social housing was associated with significantly higher odds of being a smoker across men and women of all ages, this link was stronger among women compared with men. This may be an indication that women who live in social housing may be more likely than men to be experiencing other disadvantages (e.g. being unemployed or a single parent) which compound their greater likelihood of smoking (14). The disparity in smoking prevalence was also more pronounced among over-35s compared with those aged 16-34y. In addition, the association between living in social housing and higher levels of addiction was strongest among the youngest age group (16-34y), with no significant difference in level of addiction by housing tenure observed in the oldest age group (≥65y). These findings suggest that living in social housing may be associated with greater risk of people who take up smoking at younger ages continuing to smoke throughout the lifecourse. Younger adults who live in social housing are more likely to smoke than those who live in other housing types and, in particular, have higher levels of addiction, which make it harder for them to quit. This results in a greater disparity in smoking prevalence at older ages, as younger smokers outside social housing who have lower levels of addiction may quit with less difficulty before they reach middle age.

Without targeted action, smoking-related disparities are likely to have significant implications for the health of people and their families living in social housing. The adverse effects of smoking on health and life expectancy are well established, and the transmission to the next generation (15), but much of the harm caused by smoking can be reversed by quitting (16,17). This offers huge policy potential to 'level up' and reduce the damage smoking causes. Various approaches have been suggested to better support smokers in social housing, including ways in which social landlords can maximise their opportunity to improve tenants' wellbeing (7). Most recently, the All Party Parliamentary Group on Smoking and Health recommended an at-scale intervention to provide free e-cigarettes and behavioural support to smokers in social housing (18) based on a successful pilot in Salford in the North of England (19). We note that tobacco control measures often work synergistically and targeted policies are likely to be most effective in the context of a comprehensive, integrated approach (18,20). Given the particularly high levels of addiction among younger smokers living in social housing and high prevalence of smoking at older ages, addressing uptake of smoking is an important target. Studies have shown that raising the age of sale can be effective in narrowing inequalities in initiation of smoking (21,22).

For peer review only - http://bmjopen.bmj.com/site/about/guidelines.xhtml

A major strength of this study was the large, representative sample. There were also several limitations. First, all outcomes were self-reported, introducing scope for bias. Measurement of quit attempts and use of support relied on recall of the past year and quit success was not biochemically verified. While the latter would be a significant limitation in randomised trials (because smokers who receive active treatment may feel social pressure to claim abstinence), social pressure and the associated rate of misreporting is low in population surveys (23). Moreover, we would not expect the extent of misreporting to differ by housing tenure meaning our results are unlikely to materially be affected. Secondly, while we adjusted for key sociodemographic variables, it is possible there was residual confounding by unmeasured variables, such as mental or physical health problems. Thirdly, the data were collected in England and the findings may not generalise to other countries with different approaches to social housing or tobacco control.

In conclusion, there remain stark inequalities in smoking and quitting behaviour by housing tenure in England, with declines in prevalence stalling between 2015 and 2020 despite progress in the rest of the population. In the absence of targeted interventions to boost quitting among social housing residents, inequalities in health are likely to worsen. In the context of the UK Government's commitment to levelling up, tackling smoking in social housing should be an urgent priority.

For peer review only

For peer review only - http://bmjopen.bmj.com/site/about/guidelines.xhtml

Figure legends

Figure 1. Annual smoking prevalence among adults in England living in social housing compared with other housing tenures, January 2015 through February 2020. Shaded bands indicate 95% confidence intervals. Bases (weighted n): social housing 2015 n=2849, 2016 n=2910, 2017 n=2717, 2018 n=2579, 2019 n=2420, 2020 n=373; other housing 2015 n=17132, 2016 n=17520, 2017 n=17662, 2018 n=18106, 2019 n=18215, 2020 n=3029. *Note: Data for 2020 are from January and February only.

For peer review only

For peer review only - http://bmjopen.bmj.com/site/about/guidelines.xhtml

Declarations

Competing interests

JB has received unrestricted research funding from Pfizer, who manufacture smoking cessation medications. All authors declare no financial links with tobacco companies or e-cigarette manufacturers or their representatives.

Funding

This work was supported by Cancer Research UK (C1417/A22962).

Author contributions

SJ, HC, DA, RT and JB conceived and designed the study. SJ analysed the data and wrote the first draft. All authors provided critical revisions.

Data sharing

Data are available on request from the corresponding author.

For peer review only

For peer review only - http://bmjopen.bmj.com/site/about/guidelines.xhtml

References

1. Institute of Health Equity. Fair Society Healthy Lives (The Marmot Review) [Internet]. 2010 [cited 2018 Oct 23]. Available from: http://www.instituteofhealthequity.org/resources-reports/fair-society-healthy-lives-the-marmot-review

2. Hiscock R, Bauld L, Amos A, Fidler JA, Munafò M. Socioeconomic status and smoking: a review. Ann N Y Acad Sci. 2012 Feb;1248:107–23.

3. Office for National Statistics. Adult smoking habits in the UK: 2019 [Internet]. 2020. Available from: https://www.ons.gov.uk/peoplepopulationandcommunity/healthandsocialcare/healthandlifeexpectancies/bulletins/adultsmokinghabitsingreatbritain/2019#the-proportion-who-are-current-smokers-in-the-uk-its-consistent-countries-and-local-areas-2011-to-2019

4. Jarvis MJ, Wardle J. Social patterning of individual health behaviours: the case of cigarette smoking. Soc Determinants Health. 1999;2:224–37.

5. Beard E, Brown J, Jackson SE, West R, Kock L, Boniface S, et al. Independent associations between different measures of socioeconomic position and smoking status: A cross-sectional study of adults in England. Nicotine Tob Res [Internet]. 2020 [cited 2020 Mar 19]; Available from: https://academic.oup.com/ntr/advance-article/doi/10.1093/ntr/ntaa030/5728574

6. Jackson SE, Smith C, Cheeseman H, West R, Brown J. Finding smoking hot-spots: a cross-sectional survey of smoking patterns by housing tenure in England. Addiction. 2019;114(5):889–95.

7. Action on Smoking and Health. Smoking in the home: New solutions for a Smokefree Generation [Internet]. 2018 [cited 2019 Sep 4]. Available from: http://ash.org.uk/wp-content/uploads/2018/11/FINAL-2018-Smokefree-Housing-report-web.pdf

8. Department of Health. Towards a Smokefree Generation: A Tobacco Control Plan for England [Internet]. London: Department of Health; 2017 Jul [cited 2018 Sep 13]. Available from: https://assets.publishing.service.gov.uk/government/uploads/system/uploads/attachment_data/file/630217/Towards_a_Smoke_free_Generation_-_A_Tobacco_Control_Plan_for_England_2017-2022__2_.pdf

9. HM Treasury. Build Back Better: our plan for growth [Internet]. 2021 [cited 2021 Aug 2]. Available from: https://assets.publishing.service.gov.uk/government/uploads/system/uploads/attachment_data/file/968403/PfG_Final_Web_Accessible_Version.pdf

10. Fidler JA, Shahab L, West O, Jarvis MJ, McEwen A, Stapleton JA, et al. "The smoking toolkit study": a national study of smoking and smoking cessation in England. BMC Public Health. 2011 Jun 18;11:479.

11. Jackson SE, Beard E, Kujawski B, Sunyer E, Michie S, Shahab L, et al. Comparison of Trends in Self-reported Cigarette Consumption and Sales in England, 2011 to 2018. JAMA Netw Open. 2019 Aug 2;2(8):e1910161.

12. Kotz D, Brown J, West R. Predictive validity of the Motivation To Stop Scale (MTSS): A single-item measure of motivation to stop smoking. Drug Alcohol Depend. 2013 Feb 1;128(1):15–9.

For peer review only - http://bmjopen.bmj.com/site/about/guidelines.xhtml

13. National Readership Survey. Social grade - definitions and discriminatory power [Internet]. 2007 [cited 2012 Oct 1]. Available from: http:// www.nrs.co.uk/lifestyle.html

14. Hiscock R, Bauld L, Amos A, Platt S. Smoking and socioeconomic status in England: the rise of the never smoker and the disadvantaged smoker. J Public Health Oxf Engl. 2012 Aug;34(3):390–6.

15. Leonardi-Bee J, Jere ML, Britton J. Exposure to parental and sibling smoking and the risk of smoking uptake in childhood and adolescence: a systematic review and meta-analysis. Thorax. 2011 Oct 1;66(10):847–55.

16. Doll R, Peto R, Boreham J, Sutherland I. Mortality in relation to smoking: 50 years' observations on male British doctors. BMJ. 2004 Jun 24;328(7455):1519.

17. Pirie K, Peto R, Reeves GK, Green J, Beral V. The 21st century hazards of smoking and benefits of stopping: a prospective study of one million women in the UK. The Lancet. 2013 Jan 12;381(9861):133–41.

18. APPG on Smoking and Health. Delivering a Smokefree 2030: The All Party Parliamentary Group on Smoking and Health recommendations for the Tobacco Control Plan 2021 [Internet]. London; 2021 [cited 2021 Dec 13]. Available from: https://ash.org.uk/about-ash/all-party-parliamentary-group-on-smoking-health/inquiries-reports/deliveringasf2030appgtcp2021/

19. Greater Manchester Combined Authority, NHS in Greater Manchester, University of Salford, Salford City Council. E-cigarette swap to stop evaluation summary [Internet]. Manchester, UK; 2020 [cited 2021 Dec 13]. Available from: https://www.gmhsc.org.uk/wp-content/uploads/2020/08/E-cig-Eval-Summary19_FINAL.pdf

20. Khan J. The Khan review: making smoking obsolete [Internet]. Office for Health Improvement and Disparities; 2022 Jun [cited 2022 Jun 15]. Available from: https://www.gov.uk/government/publications/the-khan-review-making-smoking-obsolete

21. Colston DC, Xie Y, Patrick ME, Thrasher JF, Titus AR, Elliott MR, et al. Tobacco 21 laws may reduce smoking and tobacco-related health disparities among youth in the U.S. Prev Med Rep. 2022 Jun;27:101762.

22. Anyanwu PE, Craig P, Katikireddi SV, Green MJ. Impact of UK Tobacco Control Policies on Inequalities in Youth Smoking Uptake: A Natural Experiment Study. Nicotine Tob Res. 2020 Oct 29;22(11):1973–80.

23. West R, Zatonski W, Przewozniak K, Jarvis MJ. Can we trust national smoking prevalence figures? Discrepancies between biochemically assessed and self-reported smoking rates in three countries. Cancer Epidemiol Biomark Prev Publ Am Assoc Cancer Res Cosponsored Am Soc Prev Oncol. 2007 Apr;16(4):820–2.

For peer review only - http://bmjopen.bmj.com/site/about/guidelines.xhtml

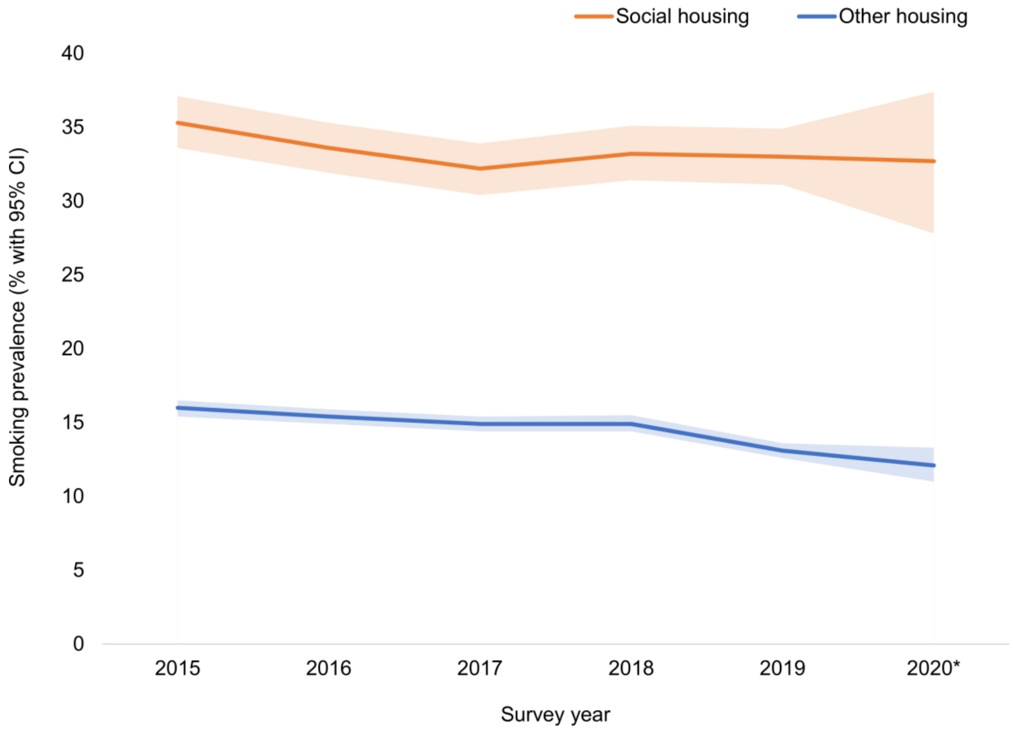

Figure 1. Annual smoking prevalence among adults in England living in social housing compared with other housing tenures, January 2015 through February 2020. Shaded bands indicate 95% confidence intervals. Bases (weighted n): social housing 2015 n=2849, 2016 n=2910, 2017 n=2717, 2018 n=2579, 2019 n=2420, 2020 n=373; other housing 2015 n=17132, 2016 n=17520, 2017 n=17662, 2018 n=18106, 2019 n=18215, 2020 n=3029. *Note: Data for 2020 are from January and February only.

188x135mm (300 x 300 DPI)

Smoking in social housing among adults in England, 2015-2020: a nationally representative survey

Sarah E. Jackson[1,2], Hazel Cheeseman[3], Deborah Arnott[3], Robbie Titmarsh[3], Jamie Brown[1,2]

[1] Department of Behavioural Science and Health, University College London, London, UK

[2] SPECTRUM Consortium, UK

[3] Action on Smoking and Health, London, UK

Supplementary material

Supplementary Table 1. Interactions between housing tenure and age group

Supplementary Table 2. Interactions between housing tenure and sex

Supplementary Table 3. Association between use of evidence-based support and cessation among past-year smokers who made a quit attempt: interaction with housing tenure

Supplementary Table 4. Sensitivity analysis using log-binomial regression to test associations between housing tenure and smoking and cessation behaviour

For peer review only

Supplementary Table 1. Interactions between housing tenure and age group

	35-64 (vs. 16-34)			≥65 (vs. 16-34)		
	OR/B*	95% CI	*p*	OR/B	95% CI	*p*
All adults						
% Cigarette smokers	1.42	1.30 to 1.55	<0.001	1.37	1.20 to 1.56	<0.001
Current cigarette smokers						
Mean cigarettes per day	-0.97	-1.53 to -0.41	0.001	-1.86	-2.75 to -0.96	<0.001
% First smoke within 30 min of waking	0.86	0.74 to 1.00	0.043	0.59	0.47 to 0.74	<0.001
% High motivation to stop	1.00	0.82 to 1.22	0.994	0.90	0.61 to 1.34	0.611
% Regular exposure to smoking by others	1.22	1.04 to 1.43	0.015	1.48	1.17 to 1.88	0.001
Past-year smokers						
% Past year quit attempt	1.15	0.99 to 1.33	0.069	1.02	0.78 to 1.32	0.912
Past year quit attempt						
% Not currently smoking	0.92	0.64 to 1.32	0.645	1.14	0.60 to 2.17	0.682
% Used any cessation support**	0.77	0.60 to 0.99	0.037	0.79	0.50 to 1.24	0.299
% Used behavioural support	0.62	0.26 to 1.49	0.284	0.37	0.11 to 1.24	0.107
% Used NRT OTC	0.74	0.51 to 1.07	0.112	1.17	0.64 to 2.12	0.616
% Used e-cigarettes	0.84	0.65 to 1.08	0.177	0.73	0.42 to 1.26	0.261
% Used prescription medication	0.98	0.60 to 1.60	0.930	0.83	0.39 to 1.79	0.637

* OR/B adjusted for sex, age, social grade, government office region, and survey year. **Any cessation support includes behavioural support, nicotine replacement therapy (NRT) bought over-the-counter (OTC), e-cigarettes, and prescription medication.

For peer review only

Supplementary Table 2. Interactions between housing tenure and sex

	Female (vs. male)		
	OR/B*	95% CI	*p*
All adults			
% Cigarette smokers	1.11	1.02 to 1.20	0.014
Current cigarette smokers			
Mean cigarettes per day	-0.11	-0.64 to 0.42	0.678
% First smoke within 30 min of waking	1.02	0.89 to 1.17	0.805
% High motivation to stop	0.90	0.74 to 1.09	0.283
% Regular exposure to smoking by others	0.93	0.80 to 1.07	0.302
Past-year smokers			
% Past year quit attempt	0.97	0.84 to 1.11	0.640
Past year quit attempt			
% Not currently smoking	1.02	0.72 to 1.45	0.901
% Used any cessation support**	1.23	0.97 to 1.56	0.093
% Used behavioural support	0.82	0.39 to 1.74	0.605
% Used NRT OTC	1.13	0.80 to 1.61	0.486
% Used e-cigarettes	1.14	0.89 to 1.47	0.308
% Used prescription medication	1.13	0.74 to 1.74	0.571

* OR/B adjusted for sex, age, social grade, government office region, and survey year. **Any cessation support includes behavioural support, nicotine replacement therapy (NRT) bought over-the-counter (OTC), e-cigarettes, and prescription medication.

For peer review only

Supplementary Table 3. Association between use of evidence-based support and cessation among past-year smokers who made a quit attempt: interaction with housing tenure

	Not currently smoking		
	OR*	95% CI	*p*
Used any cessation support**	1.11	0.94-1.30	0.218
Housing tenure	0.62	0.46-0.84	0.002
Used any cessation support x housing tenure (interaction)	0.93	0.64-1.34	0.684

*OR adjusted for sex, age, social grade, government office region, survey year, mean cigarettes per day, and smoking first cigarette within 30 minutes of waking.

**Any cessation support includes behavioural support, nicotine replacement therapy (NRT) bought over-the-counter (OTC), e-cigarettes, and prescription medication.

For peer review only

Supplementary Table 4. Sensitivity analysis using log-binomial regression to test associations between housing tenure and smoking and cessation behaviour

	Social housing	Other housing	Unadjusted			Adjusted*		
			RR	95% CI	*p*	RR	95% CI	*p*
**All adults**								
% Cigarette smokers	33.5	14.8	2.29	2.21 to 2.37	<0.001	1.73	1.66 to 1.80	<0.001
**Current cigarette smokers**								
% First smoke within 30 min of waking	57.4	42.6	1.34	1.27 to 1.42	<0.001	1.20	1.13 to 1.28	<0.001
% High motivation to stop	14.7	15.0	0.97	0.89 to 1.07	0.555	1.02	0.93 to 1.13	0.689
% Regular exposure to smoking by others	68.4	68.6	1.01	0.96 to 1.06	0.854	1.01	0.96 to 1.07	0.687
**Past-year smokers**								
% Past-year quit attempt	32.4	30.9	1.06	0.99 to 1.13	0.088	1.11	1.03 to 1.19	0.005
**Past-year quit attempt**								
% Not currently smoking	11.6	18.9	0.62	0.52 to 0.73	<0.001	0.69	0.58 to 0.82	<0.001
% Used any cessation support**	59.0	54.4	1.10	1.00 to 1.20	0.051	1.09	0.99 to 1.21	0.086
% Used behavioural support	2.8	2.2	1.27	0.90 to 1.79	0.180	1.27	0.86 to 1.87	0.237
% Used NRT OTC	13.4	13.0	1.06	0.90 to 1.24	0.507	0.91	0.76 to 1.09	0.305
% Used e-cigarettes	33.9	32.1	1.06	0.95 to 1.18	0.319	1.11	0.98 to 1.25	0.103
% Used prescription medication	9.0	7.1	1.28	1.05 to 1.56	0.015	1.33	1.06 to 1.66	0.014

*RR adjusted for sex, age, social grade, government office region, and survey year.

**Any cessation support includes behavioural support, nicotine replacement therapy (NRT) bought over-the-counter (OTC), e-cigarettes, and prescription medication.

For peer review only

STROBE Statement—Checklist of items that should be included in reports of ***cross-sectional studies***

	Item No	Recommendation	Page No
**Title and abstract**	1	(*a*) Indicate the study's design with a commonly used term in the title or the abstract	1
		(*b*) Provide in the abstract an informative and balanced summary of what was done and what was found	2
**Introduction**			
Background/rationale	2	Explain the scientific background and rationale for the investigation being reported	4
Objectives	3	State specific objectives, including any prespecified hypotheses	4
**Methods**			
Study design	4	Present key elements of study design early in the paper	4
Setting	5	Describe the setting, locations, and relevant dates, including periods of recruitment, exposure, follow-up, and data collection	4
Participants	6	(*a*) Give the eligibility criteria, and the sources and methods of selection of participants	4
Variables	7	Clearly define all outcomes, exposures, predictors, potential confounders, and effect modifiers. Give diagnostic criteria, if applicable	5
Data sources/ measurement	8*	For each variable of interest, give sources of data and details of methods of assessment (measurement). Describe comparability of assessment methods if there is more than one group	5
Bias	9	Describe any efforts to address potential sources of bias	6
Study size	10	Explain how the study size was arrived at	6
Quantitative variables	11	Explain how quantitative variables were handled in the analyses. If applicable, describe which groupings were chosen and why	5
Statistical methods	12	(*a*) Describe all statistical methods, including those used to control for confounding	6
		(*b*) Describe any methods used to examine subgroups and interactions	6
		(*c*) Explain how missing data were addressed	6
		(*d*) If applicable, describe analytical methods taking account of sampling strategy	6
		(*e*) Describe any sensitivity analyses	6
**Results**			
Participants	13*	(a) Report numbers of individuals at each stage of study—eg numbers potentially eligible, examined for eligibility, confirmed eligible, included in the study, completing follow-up, and analysed	6
		(b) Give reasons for non-participation at each stage	n/a
		(c) Consider use of a flow diagram	n/a
Descriptive data	14*	(a) Give characteristics of study participants (eg demographic, clinical, social) and information on exposures and potential confounders	7
		(b) Indicate number of participants with missing data for each variable of interest	9
Outcome data	15*	Report numbers of outcome events or summary measures	9
Main results	16	(*a*) Give unadjusted estimates and, if applicable, confounder-adjusted estimates and their precision (eg, 95% confidence interval). Make clear which confounders were adjusted for and why they were included	9

For peer review only

		(*b*) Report category boundaries when continuous variables were categorized	n/a
		(*c*) If relevant, consider translating estimates of relative risk into absolute risk for a meaningful time period	n/a
Other analyses	17	Report other analyses done—eg analyses of subgroups and interactions, and sensitivity analyses	10
**Discussion**			
Key results	18	Summarise key results with reference to study objectives	11
Limitations	19	Discuss limitations of the study, taking into account sources of potential bias or imprecision. Discuss both direction and magnitude of any potential bias	12
Interpretation	20	Give a cautious overall interpretation of results considering objectives, limitations, multiplicity of analyses, results from similar studies, and other relevant evidence	11-12
Generalisability	21	Discuss the generalisability (external validity) of the study results	12
**Other information**			
Funding	22	Give the source of funding and the role of the funders for the present study and, if applicable, for the original study on which the present article is based	15

*Give information separately for exposed and unexposed groups.

Note: An Explanation and Elaboration article discusses each checklist item and gives methodological background and published examples of transparent reporting. The STROBE checklist is best used in conjunction with this article (freely available on the Web sites of PLoS Medicine at http://www.plosmedicine.org/, Annals of Internal Medicine at http://www.annals.org/, and Epidemiology at http://www.epidem.com/). Information on the STROBE Initiative is available at www.strobe-statement.org.

For peer review only

For peer review only - http://bmjopen.bmj.com/site/about/guidelines.xhtml
